# Lymph Node Isolated Tumor Cells in Patients With Endometrial Cancer

**DOI:** 10.1001/jamanetworkopen.2024.0988

**Published:** 2024-03-18

**Authors:** Koji Matsuo, Ling Chen, Monica K. Neuman, Maximilian Klar, Joseph W. Carlson, Lynda D. Roman, Jason D. Wright

**Affiliations:** 1 Division of Gynecologic Oncology, Department of Obstetrics and Gynecology, University of Southern California, Los Angeles; 2 Norris Comprehensive Cancer Center, University of Southern California, Los Angeles; 3 Division of Gynecologic Oncology, Department of Obstetrics and Gynecology, Columbia University College of Physicians and Surgeons, New York, New York; 4 Department of Obstetrics and Gynecology, University of Freiburg Faculty of Medicine, Freiburg, Germany; 5 Department of Pathology, University of Southern California, Los Angeles

## Abstract

**Importance:**

Isolated tumor cells (ITCs) are the histopathological finding of small clusters of cancer cells no greater than 0.2 mm in diameter in the regional lymph nodes. For endometrial cancer, the prognostic significance of ITCs is uncertain.

**Objective:**

To assess clinico-pathological characteristics and oncologic outcomes associated with ITCs in endometrial cancer.

**Design, Setting, and Participants:**

This retrospective cohort study using the National Cancer Database included patients with endometrial cancer who had primary hysterectomy and nodal evaluation from 2018 to 2020. Patients with microscopic and macroscopic nodal metastases and distant metastases were excluded. Data were analyzed from June to September 2023.

**Exposure:**

Regional nodal status with ITCs (N0[i+] classification) or no nodal metastasis (N0 classification).

**Main Outcomes and Measures:**

(1) Clinical and tumor characteristics associated with ITCs, assessed with multivariable binary logistic regression model, and (2) overall survival (OS) associated with ITCs, evaluated by nonproportional hazard analysis with restricted mean survival time at 36 months.

**Results:**

A total of 56 527 patients were included, with a median (IQR) age of 64 (57-70) years. The majority had T1a lesion (37 836 [66.9%]) and grade 1 or 2 endometrioid tumors (40 589 [71.8%]). ITCs were seen in 1462 cases (2.6%). In a multivariable analysis, ITCs were associated with higher T classification, larger tumor size, lymphovascular space invasion (LVSI), and malignant peritoneal cytology. Of those tumor factors, LVSI had the largest association with ITCs (7.9% vs 1.4%; adjusted odds ratio [aOR], 4.37; 95% CI, 3.87-4.93), followed by T1b classification (5.3% vs 1.3%; aOR, 2.62; 95% CI, 2.30-2.99). At the cohort level, 24-month OS rates were 94.3% (95% CI, 92.4%-95.7%) for the ITC group and 96.1% (95% CI, 95.9%-96.3%) for the node-negative group, and the between-group difference in expected mean OS time at 36 months was 0.35 (SE, 0.19) months, but it was not statistically significant (*P* = .06). There was a statistically significant difference in OS when the low-risk group (stage IA, grade 1-2 endometrioid tumors with no LVSI) was assessed per nodal status and adjuvant therapy use (*P* < .001): (1) among the cases treated with surgical therapy alone, 24-month OS rates were 95.9% (95% CI, 89.5%-98.5%) for the ITC group and 98.8% (95% CI, 98.6%-99.0%) for the node-negative group, and the between-group mean OS time difference at 36 months was 0.61 (SE, 0.43) months (*P* = .16); and (2) among the cases with ITCs, adjuvant therapy (radiotherapy alone, systemic chemotherapy alone, or both) was associated with improved survival compared with no adjuvant therapy (24-month OS rates, 100% vs 95.9%; between-group mean OS time difference at 36 months, 0.95 [SE, 0.43] months; *P* = .03).

**Conclusions and Relevance:**

In this cohort study of patients with surgically staged endometrial cancer, the results of exploratory analysis suggested that presence of ITCs in the regional lymph node may be associated with OS in the low-risk group. While adjuvant therapy was associated with improved OS in the low-risk group with ITCs, careful interpretation is necessary given the favorable outcomes regardless of adjuvant therapy use. This hypothesis-generating observation in patients with low-risk endometrial cancer warrants further investigation, especially with prospective setting.

## Key Points

**Question:**

Are isolated tumor cells (ITCs) in regional lymph nodes of patients with endometrial cancer associated with overall survival (OS)?

**Findings:**

In this cohort study of 56 527 patients with endometrial cancer, higher T classification, larger tumor size, lymphovascular space invasion, and malignant peritoneal cytology were associated with the presence of ITCs. OS was different in the low-risk group (stage IA, grade 1-2 endometrioid tumors with absence of lymphovascular space invasion) based on ITCs and adjuvant therapy use; adjuvant therapy for patients with ITCs was associated with improved OS compared with nonuse.

**Meaning:**

These findings suggest that ITCs may be associated with OS in low-risk endometrial cancer; careful interpretation and validation are necessary for adjuvant therapy use in low-risk endometrial cancer with ITCs.

## Teaser

This cohort study assesses clinic-pathological characteristics and oncologic outcomes associated with isolated tumor cells among US patients with endometrial cancer.

## Introduction

Endometrial cancer is a malignant neoplasm arising in the uterine corpus that was the fourth most common cancer in women in the United States in 2023.^1^ Oncologic outcomes and management of patients with endometrial cancer are largely dependent on the extent of tumor spread, and the status of the regional lymph nodes confers important prognostic information and allows tailored adjuvant treatment.^2^

Isolated tumor cells (ITCs) are the histopathological finding of a small cluster of cancer cells with a diameter of no greater than 0.2 mm or 200 cells in the regional lymph nodes.^3^ According to the National Comprehensive Cancer Network Clinical Practice Guidelines, ITCs are a relatively new entity in endometrial cancer, and their prognostic significance and therapeutic implications are currently under active investigation.^4^

Available studies on ITCs in endometrial cancer are limited by modest sample sizes (median, 31 cases; range, 18-175 cases) and short duration of follow-up (median, 2.3 years; range, 2.1-2.6 years) across all studies (eTable 1 in Supplement 1).^5,6,7,8,9^ In clinical practice in the United States, despite the lack of clear consensus and recommendations, health care practitioners frequently offer adjuvant therapy for patients with ITCs identified in the regional lymph nodes.^9^ This trend in the use of adjuvant therapy includes patients with tumors with an otherwise low risk of recurrence for whom prognosis is generally favorable and adjuvant therapy has historically not been indicated.^4,9,10^

Several investigators have previously suggested that ITCs are not prognostic and adjuvant therapy based on the presence of ITCs alone may not improve survival.^5,7,11^ Thus, the objective of the current study was to assess clinico-pathological characteristics and oncologic outcomes associated with ITCs in patients with endometrial cancer in a large, nationwide population.

## Methods

### Data and Setting

This retrospective cohort study used the National Cancer Database (NCDB). The NCDB program is a nationwide cancer registry in the United States that collects data from Commission on Cancer (CoC)–accredited facilities.^12^ Through the joint mechanism of the American College of Surgeons and the American Cancer Society, more than 1500 CoC-accredited facilities participate in this tumor registry. NCDB captures approximately 70% of all new invasive cancers in the United States in each year. The Columbia University institutional review board deemed the study nonhuman participant research, as it included only publicly available, deidentified data; therefore, the requirement for informed consent was waived. The data are reported in accord with the Strengthening the Reporting of Observational Studies in Epidemiology (STROBE) reporting guideline for cohort studies.^13^

### Inclusion and Exclusion Criteria

Eligible patients for the study were women with a diagnosis of endometrial cancer who underwent primary hysterectomy and nodal evaluation from 2018 to 2020. Histologic types included grade 1 and 2 endometrioid, grade 3 endometrioid, serous, clear cell, carcinosarcoma, undifferentiated, and mixed tumors.^14^ We chose 2018 as the initial year of analysis due to the introduction of information on ITCs in the program.^3^ Identification of the cases followed the World Health Organization’s *International Classification of Disease for Oncology*, 3rd edition, and the program’s surgical coding schema (eTable 2 in Supplement 1).

Exclusion criteria were nodal metastasis including microscopic and macroscopic nodal metastases. Cases with American Joint Commission on Cancer (AJCC) M1 or unknown M classification, AJCC T4 or unknown T classification, no or unknown hysterectomy, no or unknown nodal evaluation at surgery, lack of pathologic diagnostic confirmation, neoadjuvant therapy prior to hysterectomy, and patients with a history of other malignant neoplasm were also excluded.

### Exposure

Cases that met the inclusion and exclusion criteria were classified based on the status of the regional lymph nodes. Cases with AJCC N0(i+) classification were grouped as the ITC group, and cases with AJCC N0 classification were grouped as the node-negative group.

### Main Outcomes and Measures

The main outcomes were (1) clinical and tumor characteristics associated with ITCs and (2) overall survival (OS) associated with ITCs. OS was defined as the time interval from the date of diagnosis of endometrial cancer until death from any cause (all-cause death). Survival information in NCDB is updated annually. Patients alive at last follow-up were censored.

Secondary outcomes included use of adjuvant therapy following hysterectomy. Adjuvant therapy included pelvic radiotherapy (external beam radiotherapy [EBRT] or vaginal brachytherapy) and systemic chemotherapy.

### Study Covariates

Patient demographics included age (<60, 60-69, and ≥70 years), year of endometrial cancer diagnosis (2018, 2019, and 2020), race and ethnicity (Black, Hispanic, other [including American Indian or Alaska Native and Asian patients and patients with unknown race and ethnicity], and White) determined by the program, primary payer (private, Medicaid, Medicare, uninsured, and other government), socioeconomic status as defined by the NCDB (high, medium high, medium low, and low), residential status as defined by the NCDB (metropolitan, urban, and rural), and Charlson-Deyo comorbidity index (0, 1, and ≥2) scored by the program. Race and ethnicity were assessed in this study as these factors are associated with the incidence, characteristics, and outcome of endometrial cancer.

Facility data included type (academic or research, integrated network, comprehensive community, or community) and regional location in the United States (Northeast, Midwest, South, and West). Surgical data included use of sentinel lymph node (SLN) biopsy for nodal evaluation. Tumor characteristics included histology as described previously, AJCC T classification (T1a, T1b, T1 not otherwise specified, T2, and T3) and tumor size (≤4 or >4 cm) grouped per prior analysis,^9^ lymphovascular space invasion (LVSI; present or absent), and peritoneal cytology status (malignant including atypical or negative). Missing data were classified as unknown and were included in the analysis.

### Statistical Analysis

We assessed the clinico-pathological characteristics associated with ITCs. A multivariable binary logistic regression model was fitted for this step of the analysis, and the effect size for ITCs was expressed with adjusted odds ratio (aOR) with a corresponding 95% CI.

We assessed the association between use of adjuvant therapy and ITCs. Frequency tables were compiled to assess radiotherapy and chemotherapy utilization based on the presence of ITCs and compared using χ^2^ tests.

To assess the association between the presence of ITCs and survival, we fitted a model for restricted mean survival time (RMST).^15^ Time-to-event from the endometrial cancer diagnosis to death from any cause at 36 months was examined. An RMST model was selected due to the crossing survival curves between the 2 exposure groups. The time-to-event point of 36 months to assess the between-group difference was chosen due to relatively limited follow-up, in that survival information was available for the 2018 to 2019 period and the data were released in October 2022.

In the RMST model, the exposure-outcome association was further adjusted for all the measured study covariates listed previously. This approach was to account for possible association either to the exposure or outcome measures. The Šídák adjustment was used for multiple comparisons in more than 2 groups. Survival curves were constructed with the Kaplan-Meier method, and 24-month OS rates were estimated with corresponding 95% CIs. This time point was preselected due to the median follow-up of the study cohort. OS was also assessed with RMST based on the use of adjuvant therapy in the exposure groups. Use of any adjuvant therapy was aggregated to comply with data use guidelines to avoid small cell sizes.

We performed a number of sensitivity analyses for patient groups based on risk factors. We evaluated low-risk endometrial cancer, patients who have an excellent prognosis with surgery alone. This group included stage IA, grade 1 or 2 endometrioid endometrial cancer without LVSI.^10^ Other subgroups included aggressive or advanced tumor groups with high-risk histology (grade 3 endometrioid, serous, clear cell, carcinosarcoma, undifferentiated, and mixed)^14^ and AJCC T2 and T3 classifications. These patients are at greater risk for recurrence and generally receive adjuvant therapy after hysterectomy.^4,10^

All statistical tests were 2-sided, and a *P* < .05 was considered statistically significant. SAS version 9.4 (SAS Institute), including RMSTREG and LIFETEST procedures, was used for analysis. The analysis was performed from June to September 2023.

## Results

### Cohort Characteristics

**Figure 1.**
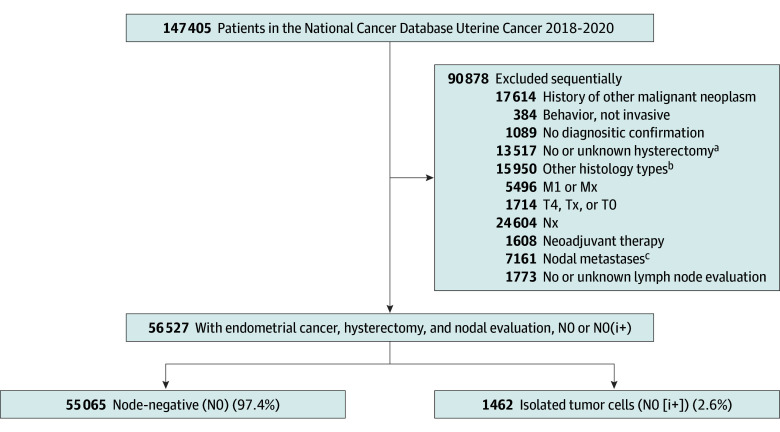
Patient Selection Schema ^a^Also excluded palliative hysterectomy. ^b^Histology subtypes other than study defined were grade 1 to 2 endometrioid, grade 3 endometrioid, serous, clear cell, carcinosarcoma, undifferentiated, and mixed. ^c^Including micrometastasis and macrometastasis.

A total of 56 527 patients with AJCC T1 to T3 and N0 or N0(i+) and M0 endometrial cancer who underwent primary hysterectomy and nodal evaluation were examined for analysis (Figure 1). The median (IQR) age of the cohort was 64 (57-70) years (Table 1). There were 5537 (9.8%) Black participants, 4402 (7.8%) Hispanic participants, and 42 828 (75.8%) White participants. More than one-third had SLN biopsy for nodal evaluation (19 910 [35.2%]). Most tumors were AJCC T1a lesions (37 836 [66.9%]) and grade 1 to 2 endometrioid histology (40 589 [71.8%]).

**Table 1.** Clinico-Pathological Characteristics Associated With ITCs

Characteristic	Patients, No. (%)	Patients with ITC, %^a^	aOR (95% CI)^b^
No.	56 527 (100.0)	2.6	NA
Age, y			
<60	18 879 (33.4)	2.3	1 [Reference]
60-69	22 019 (39.0)	2.8	1.14 (0.99-1.31)
≥70	15 629 (27.6)	2.6	0.90 (0.75-1.08)
Year			
2018	16 969 (30.0)	2.3	1 [Reference]
2019	19 866 (35.1)	2.6	1.15 (1.01-1.32)^c^
2020	19 692 (34.8)	2.8	1.21 (1.06-1.38)^c^
Race and ethnicity			
Black	5537 (9.8)	1.8	0.79 (0.63-0.98)^c^
Hispanic	4402 (7.8)	2.3	0.98 (0.79-1.23)
Other^d^	3760 (6.7)	2.9	1.14 (0.92-1.41)
White	42 828 (75.8)	2.7	1 [Reference]
Payer type			
Private	25 754 (45.6)	2.5	1 [Reference]
Medicaid	3594 (6.4)	2.8	1.04 (0.83-1.30)
Medicare	24 828 (43.9)	2.6	0.92 (0.80-1.06)
Uninsured	1326 (2.3)	2.9	1.07 (0.76-1.50)
Other government^e^	1025 (1.8)	2.3	1.00 (0.66-1.52)
Socioeconomic status			
High	8576 (15.2)	2.6	1.01 (0.84-1.21)
Medium high	15 698 (27.8)	2.7	1.01 (0.86-1.17)
Medium low	8169 (14.5)	2.5	0.92 (0.77-1.10)
Low	15 560 (27.5)	2.5	1 [Reference]
Unknown	8524 (15.1)	2.7	1.01 (0.85-1.20)
Urban rural status			
Metropolitan	46 488 (82.2)	2.6	1 [Reference]
Urban	7465 (13.2)	2.6	0.92 (0.78-1.09)
Rural	849 (1.5)	2.1	0.77 (0.47-1.25)
Unknown	1725 (3.1)	3.2	1.17 (0.88-1.56)
Facility type			
Academic or research	22 475 (39.8)	2.7	1 [Reference]
Integrated network	12 539 (22.2)	2.5	0.90 (0.78-1.04)
Comprehensive community	20 101 (35.6)	2.4	0.93 (0.82-1.05)
Community	1412 (2.5)	3.4	1.22 (0.90-1.67)
Facility location			
Northeast	11 922 (21.1)	2.9	1 [Reference]
Midwest	14 684 (26.0)	3.2	1.19 (1.03-1.39)^c^
South	19 260 (34.1)	2.1	0.79 (0.68-0.93)^c^
West	10 661 (18.9)	2.4	0.86 (0.72-1.02)
Comorbidity index			
0	41 815 (74.0)	2.6	1 [Reference]
1	10 199 (18.0)	2.7	1.10 (0.96-1.26)
≥2	4513 (8.0)	2.6	0.95 (0.78-1.17)
SLN biopsy			
No	36 617 (64.8)	2.1	1 [Reference]
Yes	19 910 (35.2)	3.5	1.86 (1.67-2.08)^c^
AJCC T classification			
T1a	37 836 (66.9)	1.3	1 [Reference]
T1b	12 429 (22.0)	5.3	2.62 (2.30-2.99)^c^
T1 NOS	459 (0.8)	NA^f^	1.00 (0.44-2.27)
T2	3131 (5.5)	4.9	2.50 (2.05-3.04)^c^
T3	2672 (4.7)	NA^f^	2.45 (1.99-3.01)^c^
Histology			
Grade 1-2 endometrioid	40 589 (71.8)	2.6	1.65 (1.30-2.08)^c^
Grade 3 endometrioid	4688 (8.3)	2.7	0.90 (0.67-1.20)
Serous	4354 (7.7)	2.0	1 [Reference]
Clear cell	825 (1.5)	NA^f^	1.00 (0.56-1.78)
Carcinosarcoma	2437 (4.3)	2.9	1.06 (0.77-1.47)
Undifferentiated	126 (0.2)	NA^f^	0.57 (0.17-1.84)
Mixed	3508 (6.2)	2.9	1.27 (0.94-1.72)
Tumor size, cm			
≤4	26 222 (46.4)	2.0	1 [Reference]
>4	15 895 (28.1)	4.2	1.46 (1.29-1.65)^c^
Unknown	14 410 (25.5)	1.9	0.95 (0.82-1.10)
LVSI			
Absent	44 179 (78.2)	1.4	1 [Reference]
Present	9798 (17.3)	7.9	4.37 (3.87-4.93)^c^
Unknown	2550 (4.5)	2.5	1.82 (1.39-2.37)^c^
Peritoneal cytology			
Negative	29 208 (51.7)	2.3	1 [Reference]
Malignant or atypical	3093 (5.5)	5.3	1.61 (1.34-1.93)^c^
Unknown	24 226 (42.9)	2.6	1.10 (0.99-1.24)

Abbreviations: AJCC, American Joint Commission on Cancer; aOR, adjusted odds ratio; ITC, isolated tumor cell; LVSI, lympho-vascular space invasion; NA, not applicable; NOS, not otherwise specified; SLN, sentinel lymph node.

^a^ Incidence rate per row level.

^b^ Multivariable binary logistic regression model.

^c^ *P* < .05.

^d^ Including American Indian or Alaska Native, Asian, and unknown.

^e^ Including unknown.

^f^ Small number suppressed.

### Clinico-Pathological Factors Associated With ITCs

ITCs were seen in 1462 cases (2.6%) overall. Clinico-pathological factors associated with an incidence of ITCs of greater than 5.0% were AJCC T1b tumors (5.3%), malignant peritoneal cytology (5.3%), and LVSI (7.9%) (Table 1).

In a multivariable analysis (Table 1), ITCs were associated with higher T classification, larger tumor size, LVSI, and malignant peritoneal cytology. Of those tumor factors, odds of having ITCs exceeded 4 times for LVSI (7.9% vs 1.4%; aOR, 4.37; 95% CI, 3.87-4.93), followed by AJCC T1b classification (5.3% vs 1.3%; aOR, 2.62; 95% CI, 2.30-2.99). Grade 1 or 2 endometrioid tumors had higher risk of ITCs compared with serous tumors (2.6% vs 2.0%; aOR, 1.65; 95% CI, 1.30-2.08).

ITCs were more commonly identified in more recent years (2.3%, 2.6%, and 2.8% for 2018, 2019, and 2020, respectively): aORs for 2019 and 2020 compared with 2018 were 1.15 (95% CI, 1.01-1.32) and 1.21 (95% CI, 1.06-1.38), respectively. Black individuals were less likely to have ITCs compared with White individuals (1.8% vs 2.7%; aOR, 0.79; 95% CI, 0.63-0.98). SLN biopsy was 86% more likely to identify ITCs compared with conventional lymphadenectomy (3.5% vs 2.1%; aOR, 1.86; 95% CI, 1.67-2.08).

### Adjuvant Therapy for ITCs

At the cohort level, patients in the ITC group were more likely to receive adjuvant therapy (72.8% vs 37.1%), including radiotherapy (42.5% vs 22.3%), chemotherapy (7.9% vs 4.5%), and both radiotherapy and chemotherapy (22.4% vs 10.3%) (*P* < .001) (eTable 3 in Supplement 1). This association with adjuvant therapy remained consistent in the low-risk group, and nearly one-fourth of patients who had lTCs received adjuvant therapy (24.9% vs 5.4%; *P* < .001) (eTable 3 in Supplement 1). ITCs were associated with increased use of EBRT (12.0% vs 0.2%) and systemic chemotherapy (8.0% vs 0.7%). In addition, utilization of combination therapy with adjuvant radiotherapy and chemotherapy was more common in the ITC group among those with high-risk histologic subtypes (43.5% vs 31.2%) and T2 to T3 tumors (43.1% vs 34.7%) (both *P* < .001) (eTable 3 in Supplement 1).

### Survival Outcomes Associated With ITCs

Survival analysis was performed for the 36 384 cases from 2018 to 2019. The median follow-up was 27 months. During the follow-up, 1740 death events occurred.

**Figure 2.**
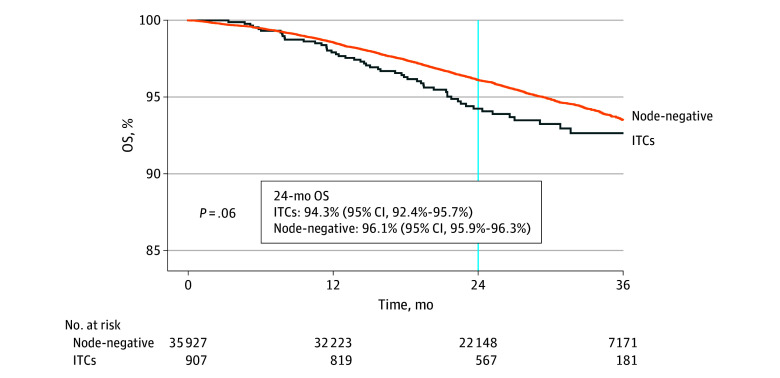
Overall Survival (OS) Based on Nodal Status Survival curves are shown for the isolated tumor cell (ITC) and node-negative groups. The vertical line indicates 24-month follow-up point. The vertical axis was truncated at 85% to 100% to maximize visibility.

At the cohort level, 24-month OS rates were 94.3% (95% CI, 92.4%-95.7%) for the ITC group and 96.1% (95% CI, 95.9%-96.3%) for the node-negative group. The between-group difference in expected mean OS time at 36 months from endometrial cancer diagnosis was 0.35 (SE, 0.19) months shorter in the ITC group than the node-negative group, but the difference was not statistically significant (*P* = .06) (Figure 2 and Table 2). After controlling for the measured study covariates, the between-group mean OS time difference at 36 months was 0.05 (95% CI, −0.32 to 0.41) months without adjuvant therapy and 0.16 (95% CI, −0.53 to 0.21) months with adjuvant therapy.

**Table 2.** Survival Outcome Associated With ITCs

Cohort	No.^a^	24-mo OS (95% CI), %^b^	RMST at 36 mo (SE)^c^	Between-group difference (SE)^c^	*P* value^d^	Between-group difference (SE)^c^	*P* value^d^
Entire cohort							
Node-negative	35 927	96.1 (95.9-96.3)	35.00 (0.03)	Reference	NA	NA	NA
ITCs	907	94.3 (92.4-95.7)	34.66 (0.18)	−0.35 (0.19)	.06	NA	NA
T1a classification^e^					.		
Node-negative, no adjuvant therapy	19 317	98.2 (98.0-98.4)	35.49 (0.03)	Reference	NA	NA	NA
ITCs, no adjuvant therapy	156	96.2 (91.1-98.4)	35.08 (0.38)	−0.42 (0.38)	.61	Reference	NA
ITCs with adjuvant therapy	149	93.9 (88.1-96.9)	34.55 (0.46)	−0.94 (0.46)	.11	−0.53 (0.59)	.75
Low-grade endometrioid^e^^,^^f^					.		
Node-negative, no adjuvant therapy	19 449	98.4 (98.2-98.6)	35.57 (0.02)	Reference	NA	NA	NA
ITCs, no adjuvant therapy	222	95.7 (91.5-97.8)	34.93 (0.36)	−0.64 (0.36)	.20	Reference	NA
ITCs, with adjuvant therapy	449	96.7 (94.3-98.1)	35.31 (0.18)	−0.26 (0.18)	.39	0.38 (0.40)	.71
T1a, low-grade endometrioid^e^^,^^f^							
Node-negative, no adjuvant therapy	16 998	98.7 (98.5-98.9)	35.65 (0.02)	Reference	NA	NA	NA
ITCs, no adjuvant therapy	147	96.0 (90.6-98.3)	35.02 (0.40)	−0.62 (0.40)	.32	Reference	NA
ITCs with adjuvant therapy	95	97.7 (90.9-99.4)	35.29 (0.42)	−0.35 (0.42)	.78	0.27 (0.58)	.95
T1a, low-grade endometrioid with no LVSI^e^^,^^f^							
Node-negative, no adjuvant therapy	15 668	98.8 (98.6-99.0)	35.65 (0.02)	Reference	NA	NA	NA
ITCs, no adjuvant therapy	117	95.9 (89.5-98.5)	35.05 (0.43)	−0.61 (0.43)	.16	Reference	NA
ITCs, with adjuvant therapy	32	100	36.00 (0)	0.35 (0.02)	<.001	0.95 (0.43)	.03
High-risk histology^g^							
Node-negative with adjuvant therapy	7086	91.5 (90.8-92.2)	33.94 (0.08)	Reference	NA	NA	NA
ITCs with adjuvant therapy	214	89.3 (84.0-92.9)	33.45 (0.50)	−0.49 (0.51)	.91	NA	NA
T2 or T3 classification							
Node-negative with adjuvant therapy	3027	91.6 (90.5-92.6)	33.94 (0.12)	Reference	NA	NA	NA
ITCs with adjuvant therapy	176	92.6 (86.9-95.9)	34.52 (0.42)	0.58 (0.44)	.71	NA	NA

Abbreviations: ITCs, isolated tumor cells; LVSI, lymphovascular space invasion; NA, not applicable; OS, overall survival; RMST, restricted mean survival time.

^a^ Cases were restricted for 2018 to 2019 due to the survival data availability.

^b^ Kaplan-Meier method was used to estimate 24-month OS rate.

^c^ Nonproportional hazard analysis with unadjusted RMST was used to estimate 36-month OS value.

^d^ Šídák adjustment for multiple comparisons (>2 groups).

^e^ The overall *P* value for T1a classification was .07; for low-grade endometrioid, .07; for T1a, low-grade endometrioid, .21; for T1a, low-grade endometrioid with no LVSI, <.001. Adjuvant therapy included postoperative radiotherapy, systemic chemotherapy, or both.

^f^ Low-grade endometrioid histology included grade 1 and 2 endometrioid types.

^g^ High-risk histology included grade 3 endometrioid, serous, clear cell, carcinosarcoma, undifferentiated, and mixed.

### Survival Outcome of ITCs in Low-Risk Patients

**Figure 3.**
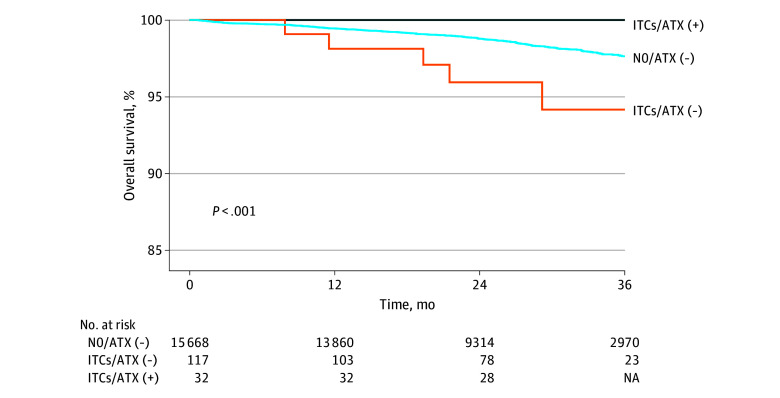
Survival Outcomes in the Low-Risk Group Survival curves are shown for isolated tumor cells (ITCs) with adjuvant therapy (ATX) (dark blue), ITCs without adjuvant therapy (orange), and node-negative without adjuvant therapy (light blue) groups. The vertical axis was truncated at 85% to 100% for maximizing the visibility. Low-risk group was defined as stage IA, grade 1 or 2 endometrioid endometrial cancer with no lymphovascular space invasion. NA indicates that a small number was suppressed; +, with; −, without.

In the low-risk group (stage IA, grade 1-2 endometrioid tumors with no LVSI; n = 15 817), there was a statistically significant difference in OS when this group was stratified for nodal status and adjuvant therapy use (*P* < .001) (Figure 3). First, in low-risk patients who underwent surgical therapy alone (hysterectomy and nodal evaluation), the 24-month OS rate was 95.9% (95% CI, 89.5%-98.5%) for the ITC group and 98.8% (95% CI, 98.6%-99.0%) for the node-negative group (117 and 15 668 patients, respectively). The between-group OS time difference at 36 months was shorter by 0.61 (SE, 0.43) months in the ITC group compared with the node-negative group (*P* = .16) (Table 2). Second, among 149 patients in the low-risk group with ITCs, 32 patients who received any modality of adjuvant therapy (radiotherapy and/or systemic chemotherapy) had statistically significantly improved OS compared with 117 patients who did not receive adjuvant therapy (24-month rates: 100% vs 95.9% [95% CI 89.5%-98.5%]; between-group mean OS time difference at 36 months, 0.95 (SE, 0.43); *P* = .03) (Table 2).

### Survival Outcomes in Aggressive and Advanced Tumors

Among the 7300 patients in the high-risk histology group who received adjuvant therapy, ITCs in the reginal lymph nodes were not associated with OS (Table 2). The results were similar among 3203 patients in the AJCC T2 and T3 classification group (Table 2).

## Discussion

These data suggest a number of important findings. First, ITCs were associated with other tumor characteristics and pathologic factors associated with nodal metastasis. Second, patients with ITCs were more likely to receive adjuvant therapy. Third, ITCs were statistically not associated with short-term OS at the cohort level. Finally, in patients with low-risk endometrial cancer with ITCs, adjuvant therapy was associated with improved short-term OS .

ITCs were rare overall, as only 1 in 38 patients with endometrial cancer who are theoretically classified as having no lymph node metastasis were identified. However, the incidence of ITCs increases to one in every 13 to 19 cases when other high-risk pathologic factors (deep myometrial invasion, LVSI, and malignant peritoneal cytology) are present. These data, together with other studies, suggest that ITCs are likely a harbinger for early tumor spread and metastasis.^9,16,17^

Among the tumor factors associated with ITCs, the risk was greatest when LVSI was observed. This historical prognostic factor of endometrial cancer is categorized as one of the high-intermediate risk factors,^10^ and substantial LVSI is associated with local and distant recurrence.^18,19^ Substantial LVSI was recently classified as a discrete group as stage IIB in the revised cancer staging schema by the International Federation of Gynecology and Obstetrics (FIGO) in 2023.^14^ It is plausible that ITCs represent early tumor dissemination outside of the uterus in those with LVSI.

During the study period, the rate of identification of ITCs increased. This finding likely reflects the increased use of SLN biopsy for endometrial cancer in the United States.^20^ Specimens from patients who receive SLN biopsy typically undergo ultrastaging, which is more likely to identify ITCs.^9,16,21^ Based on these trends, we anticipate that detection of ITCs will be increasingly frequent in the future. Decreased rates of ITCs in Black compared with White individuals may possibly be due to the decreased utilization of SLN biopsy.^20^

Perhaps the most important question in our analysis is the association between ITCs and survival. In our cohort, among patients with low-risk endometrial cancer who did not receive adjuvant therapy, there was no statistically significant association between the presence of ITCs and survival, although survival was lower in the ITC group. These findings are generally in accord with prior investigations.^5,6,7,8^ However, follow-up in our cohort and other studies remained short, and longer-term evaluation is needed.

Somewhat surprisingly, we noted that among low-risk patients with ITCs, survival was better with adjuvant therapy. Interpretation of these findings is limited by the small sample and the overall favorable outcomes of this group of patients without other adverse pathologic features.^4,10,22^ These findings clearly warrant further investigation but raise concern about the omission of adjuvant therapy in this subgroup population. Until further data are available, well-balanced shared decision-making is recommended for patients with low-risk endometrial cancer and ITCs.

We also noted that adjuvant therapy was frequently utilized in patients with ITCs. Although current data are limited, a prior multicenter observational study found no association between adjuvant therapy and survival and suggested that the omission of adjuvant therapy was reasonable.^5^ Similarly, current clinical practice guidelines recommend against adjuvant therapy.^4,10^ However, our findings of a relatively high rate of use of adjuvant therapy suggest that there is considerable uncertainty in clinical practice. It is likely that many consider ITCs in the regional lymph node as a surrogate of pathologic nodal metastatic disease. Furthermore, as described previously, survival was only modestly superior in low-risk patients with ITCs who received adjuvant therapy compared with those treated with observation.

### Limitations

We recognized several important limitations. Details of ITCs, including number of foci and anatomical location either in pelvis or para-aortic nodes, and protocol of ultrastaging were unknown. Similarly, data on some pathologic factors, such as the extent of LVSI (focal or substantial), nodal dissection laterality, and molecular abnormalities within the tumor, were not available.^18,19,23^ Recurrence data, including anatomical site (eg, nodal or distant), and cause of death (endometrial cancer or other) are unavailable in NCDB. Accuracy of SLN biopsy was not assessed. Furthermore, as described previously, the small sample size and limited follow-up limit the power of some of our subgroup analyses.

## Conclusions

Despite these limitations, our findings suggest that the association of ITCs with survival among patients with endometrial cancer is modest and needs to be interpreted in the context of other pathologic factors (eFigure in Supplement 1). The role of adjuvant therapy for low-risk endometrial cancer that would not otherwise require adjuvant therapy clearly requires further investigation. Barring more data—especially with long-term follow-up—adhering to the current uterine factor-based treatment approach is recommended.^4,10^ For the low-risk group, information to share with the patient includes both risk and benefit of observation and adjuvant therapy.

## Notes

Supplement 1. **eTable 1.** ITCs in Endometrial Cancer: Scoping Review of Literature **eTable 2.** Coding Schema **eTable 3.** Adjuvant Therapy per Nodal Status **eFigure.** Schematic View of Prognostic Significance of ITCs

Supplement 2.
Data Sharing Statement
