# Supplementary material for: Lymph Node Isolated Tumor Cells in Patients With Endometrial Cancer
Source: JAMA Netw Open. 2024 Mar 18;7(3):e240988. doi: 10.1001/jamanetworkopen.2024.0988 (PMC10949095; doi:10.1001/jamanetworkopen.2024.0988)
Supplement: Supplement 1. — eTable 1. ITCs in Endometrial Cancer: Scoping Review of Literature eTable 2. Coding Schema eTable 3. Adjuvant Therapy per Nodal Status eFigure. Schematic View of Prognostic Significance of ITCs [file jamanetwopen-e240988-s001.pdf]

Supplemental Online Content

Matsuo K, Chen L, Neuman MK, et al. Lymph node isolated tumor cells in patients with endometrial cancer. *JAMA Netw Open.* 2024;7(3):e240988. doi:10.1001/jamanetworkopen.2024.0988

eTable 1. ITCs in Endometrial Cancer: Scoping Review Of Literature

eTable 2. Coding Schema

eTable 3. Adjuvant Therapy per Nodal Status

eFigure. Schematic View of Prognostic Significance of ITCs

This supplemental material has been provided by the authors to give readers additional information about their work.

© 2024 Matsuo K et al. *JAMA Network Open.*

eTable 1. ITCs in Endometrial Cancer: Scoping Review Of Literature

Author	Year	Study design	ITCs cases	Follow-up	Oncologic outcomes
Matsuo *et al.*[1]	2022	Population-based retrospective cohort	111	n/a	Stage I specific analysis. ITCs incidence: 1.7%. ITCs were associated with larger tumor size and deep invasion. Patients with ITCs: nearly 10 times more likely to receive adjuvant therapy with variable treatment regimens (accounted for SLN biopsy).
Backes *et al.*[2]	2021	Multicenter retrospective cohort	175	2.6 years	Among recurred, median time to recur was 1.5 years. 44.4% of recurred was stage IA G1 endometrioid. Retroperitoneal / distant recurrence risk was 4.6%. Adjuvant therapy did not improve RFS.
Ghoniem *et al.*[3]	2021	Multicenter retrospective cohort	18	2.1 years	Among patients with no adjuvant therapy, 1 (5.6%) recurred.
Plante *et al.*[4]	2017	Prospective cohort	31	2.4 years	3-yr RFS for ITCs vs node-negative: 95.5% vs 87.6%. 1 (3.2%) recurred despite adjuvant therapy (stage IB, UCS).
St Clair *et al.*[5]	2016	Retrospective cohort	23	2.2 years	3-year RFS for ITCs vs node-negative: 86% vs 90%. 95.7% of ITCs cases received adjuvant therapy.

Abbreviations: ITCs, isolated tumor cells; SLN, sentinel lymph node; RFS, recurrence-free survival.

References:
1. Matsuo K, Khetan VU, Brunette LL, et al. Characterizing isolated tumor cells in regional lymph nodes of early endometrial cancer. Gynecol Oncol. 2022;165:264-269.
2. Backes FJ, Felix AS, Plante M, et al. Sentinel lymph node (SLN) isolated tumor cells (ITCs) in otherwise stage I/II endometrioid endometrial cancer: To treat or not to treat? Gynecol Oncol. 2021;161:347-352.
3. Ghoniem K, Larish AM, Dinoi G, et al. Oncologic outcomes of endometrial cancer in patients with low-volume metastasis in the sentinel lymph nodes: An international multiinstitutional study. Gynecol Oncol. 2021;162:590-598.
4. Plante M, Stanleigh J, Renaud MC, Sebastianelli A, Grondin K, Gregoire J. Isolated tumor cells identified by sentinel lymph node mapping in endometrial cancer: Does adjuvant treatment matter? Gynecol Oncol. 2017;146:240-246.
5. St Clair CM, Eriksson AG, Ducie JA, et al. Low-Volume Lymph Node Metastasis Discovered During Sentinel Lymph Node Mapping for Endometrial Carcinoma. Ann Surg Oncol. 2016;23:1653-1659.

© 2024 Matsuo K et al. *JAMA Network Open.*

eTable 2. Coding Schema

Characteristic	Codes
**Site***	
Endometrial	C54.0, C54.1, C54.2, C54.3, C54.8, C54.9, C55.9
**Histology***	
Endometrioid	8380/3, 8381/3, 8382/3, 8383/3
Serous	8441/3, 8460/3, 8461/3, 8050/3, 8260/3
Clear cell	8310/3
Carcinosarcoma	8950/3, 8951/3, 8980/3, 8981/3
Undifferentiated	8020/3
Mixed	8255/3, 8323/3
**Surgery****	
Hysterectomy	30, 31, 32, 40, 50, 60, 61, 62, 63, 64, 65, 66, 67

*World Health Organization's International Classification of Disease for Oncology, 3rd edition. ** NCDB's RX_SUMM_SURG_PRIM_SITE.

© 2024 Matsuo K et al. *JAMA Network Open.*

eTable 3. Adjuvant Therapy per Nodal Status

	Whole		T1a		Low-grade		T1a, low-grade		T1a, low-grade, LVSI (-)	
Characteristic	N0	ITCs	N0	ITCs	N0	ITCs	N0	ITCs	N0	ITCs
**All**	55065 (100)	1462 (100)	37331 (100)	505 (100)	39527 (100)	1062 (100)	28388 (100)	384 (100)	25591 (100)	225 (100)
**Treatment**										
Hyst alone	34632 (62.9)	398 (27.2)	29643 (79.4)	245 (48.5)	29921 (75.7)	340 (32.0)	26159 (92.1)	223 (58.1)	24204 (94.6)	169 (75.1)
Hyst + RT + CT	5680 (10.3)	327 (22.4)	2438 (6.5)	78 (15.4)	833 (2.1)	153 (14.4)	85 (0.3)	28 (7.3)	59 (0.2)	*
Hyst + CT	2475 (4.5)	115 (7.9)	1172 (3.1)	36 (7.1)	478 (1.2)	51 (4.8)	150 (0.5)	17 (4.4)	116 (0.5)	*
Hyst + RT	12278 (22.3)	622 (42.5)	4078 (10.9)	146 (28.9)	8295 (21.0)	518 (48.8)	1994 (7.0)	116 (30.2)	1212 (4.7)	38 (16.9)
P-value	<.001		<.001		<.001		<.001		<.001	
**Radiotherapy ****										
None	37107 (67.4)	513 (35.1)	30815 (82.5)	281 (55.6)	30399 (76.9)	391 (36.8)	26309 (92.7)	240 (62.5)	24320 (95.0)	178 (79.1)
EBRT	3155 (5.7)	388 (26.5)	590 (1.6)	105 (20.8)	1257 (3.2)	281 (26.5)	103 (0.4)	74 (19.3)	49 (0.2)	27 (12.0)
BT	12347 (22.4)	318 (21.8)	5659 (15.2)	90 (17.8)	6882 (17.4)	239 (22.5)	1922 (6.8)	53 (13.8)	1190 (4.7)	*
EBRT + BT	2416 (4.4)	243 (16.6)	247 (0.7)	29 (5.7)	974 (2.5)	151 (14.2)	49 (0.2)	17 (4.4)	29 (0.1)	*
P-value	<.001		<.001		<.001		<.001		<.001	
**Chemotherapy**										
None	46910 (85.2)	1020 (69.8)	33721 (90.3)	391 (77.4)	38216 (96.7)	858 (80.8)	28153 (99.2)	339 (88.3)	25416 (99.3)	207 (92.0)
Yes	8155 (14.8)	442 (30.2)	3610 (9.7)	114 (22.6)	1311 (3.3)	204 (19.2)	235 (0.8)	45 (11.7)	175 (0.7)	18 (8.0)
P-value	<.001		<.001		<.001		<.001		<.001	
**Combination †**										
None	34632 (62.9)	398 (27.2)	29643 (79.4)	245 (48.5)	29921 (75.7)	340 (32.0)	26159 (92.1)	223 (58.1)	24204 (94.6)	169 (75.1)
CT alone	2475 (4.5)	115 (7.9)	1172 (3.1)	36 (7.1)	478 (1.2)	51 (4.8)	150 (0.5)	17 (4.4)	116 (0.5)	*
EBRT alone	1897 (3.4)	261 (17.9)	385 (1.0)	77 (15.2)	918 (2.3)	214 (20.2)	86 (0.3)	60 (15.6)	35 (0.1)	22 (9.8)
BT alone	9008 (16.4)	221 (15.1)	3548 (9.5)	55 (10.9)	6633 (16.8)	200 (18.8)	1865 (6.6)	46 (12.0)	1152 (4.5)	14 (6.2)
EBRT + BT	1340 (2.4)	140 (9.6)	130 (0.3)	14 (2.8)	729 (1.8)	104 (9.8)	38 (0.1)	10 (2.6)	22 (0.1)	*
EBRT + CT	1258 (2.3)	127 (8.7)	205 (0.5)	28 (5.5)	339 (0.9)	67 (6.3)	17 (0.1)	14 (3.6)	14 (0.1)	*
BT + CT	3339 (6.1)	97 (6.6)	2111 (5.7)	35 (6.9)	249 (0.6)	39 (3.7)	57 (0.2)	*	38 (0.1)	*
EBRT + BT + CT	1076 (2.0)	103 (7.0)	117 (0.3)	15 (3.0)	245 (0.6)	47 (4.4)	11 (<0.1)	*	*	*
P-value	<.001		<.001		<.001		<.001		<.001	

Number with percentage per column is shown. * Small number suppressed. ** Cases with other or unknown RT were included in the analysis but suppressed due to small numbers to comply the program instruction. † Cases with RT NOS alone or RT NOS + CT were included in the analysis but suppressed due to small numbers to comply the program instruction. Low-grade histology included grade 1-2 endometrioid tumors. Abbreviations: N0, no nodal metastasis; ITCs, isolated tumor cells; LVSI, lympho-vascular space invasion; Hyst, hysterectomy; RT, adjuvant radiotherapy; CT, adjuvant chemotherapy; EBRT, external beam radiotherapy; BT, brachytherapy; and NOS, not otherwise specified.

© 2024 Matsuo K et al. *JAMA Network Open.*

eTable 3. Adjuvant therapy per nodal status (cont).

	High-risk histology		T2-T3		SLN biopsy	
Characteristic	N0	ITCs	N0	ITCs	N0	ITCs
All	15538 (100)	400 (100)	5506 (100)	297 (100)	19211 (100)	699 (100)
**Treatment**						
Hyst alone	4711 (30.3)	58 (14.5)	945 (17.2)	35 (11.8)	13134 (68.4)	183 (26.2)
Hyst + RT + CT	4847 (31.2)	174 (43.5)	1911 (34.7)	128 (43.1)	1466 (7.6)	150 (21.5)
Hyst + CT	1997 (12.9)	64 (16.0)	827 (15.0)	43 (14.5)	561 (2.9)	52 (7.4)
Hyst + RT	3983 (25.6)	104 (26.0)	1823 (33.1)	91 (30.6)	4050 (21.1)	314 (44.9)
P-value	<.001		.01		<.001	
**Radiotherapy** **						
None	6708 (43.2)	122 (30.5)	1772 (32.2)	78 (26.3)	13695 (71.3)	235 (33.6)
EBRT	1898 (12.2)	107 (26.8)	1231 (22.4)	78 (26.3)	831 (4.3)	193 (27.6)
BT	5465 (35.2)	79 (19.8)	965 (17.5)	29 (9.8)	4069 (21.2)	166 (23.7)
EBRT + BT	1442 (9.3)	92 (23.0)	1536 (27.9)	112 (37.7)	602 (3.1)	105 (15.0)
P-value	<.001		<.001		<.001	
**Chemotherapy**						
None	8694 (56.0)	162 (40.5)	2768 (50.3)	126 (42.4)	17184 (89.4)	497 (71.1)
Yes	6844 (44.0)	238 (59.5)	2738 (49.7)	171 (57.6)	2027 (10.6)	202 (28.9)
P-value	<.001		.01		<.001	
**Combination** †						
None	4711 (30.3)	58 (14.5)	945 (17.2)	35 (11.8)	13134 (68.4)	183 (26.2)
CT alone	1997 (12.9)	64 (16.0)	827 (15.0)	43 (14.5)	561 (2.9)	52 (7.4)
EBRT alone	979 (6.3)	47 (11.8)	526 (9.6)	32 (10.8)	564 (2.9)	133 (19.0)
BT alone	2375 (15.3)	21 (5.3)	507 (9.2)	*	3098 (16.1)	117 (16.7)
EBRT + BT	611 (3.9)	36 (9.0)	788 (14.3)	50 (16.8)	375 (2.0)	64 (9.2)
EBRT + CT	919 (5.9)	60 (15.0)	705 (12.8)	46 (15.5)	267 (1.4)	60 (8.6)
BT + CT	3090 (19.9)	58 (14.5)	458 (8.3)	20 (6.7)	971 (5.1)	49 (7.0)
EBRT + BT + CT	831 (5.3)	56 (14.0)	748 (13.6)	62 (20.9)	227 (1.2)	41 (5.9)
P-value	<.001		<.001		<.001	

Number with percentage per column is shown. * Small number suppressed. ** Cases with other or unknown radiotherapy were included in the analysis but suppressed due to small numbers to comply the program instruction. † Cases with RT NOS alone or RT NOS + CT were included in the analysis but suppressed due to small numbers to comply the program instruction. High-risk histology included grade 3 endometrioid, serous, clear cell, carcinosarcoma, undifferentiated, and mixed tumors. Abbreviations: N0, no nodal metastasis; ITCs, isolated tumor cells; Hyst, hysterectomy; RT, adjuvant radiotherapy; CT, adjuvant chemotherapy; EBRT, external beam radiotherapy; BT, brachytherapy; and NOS, not otherwise specified.

© 2024 Matsuo K et al. *JAMA Network Open.*

eFigure. Schematic View of Prognostic Significance of ITCs

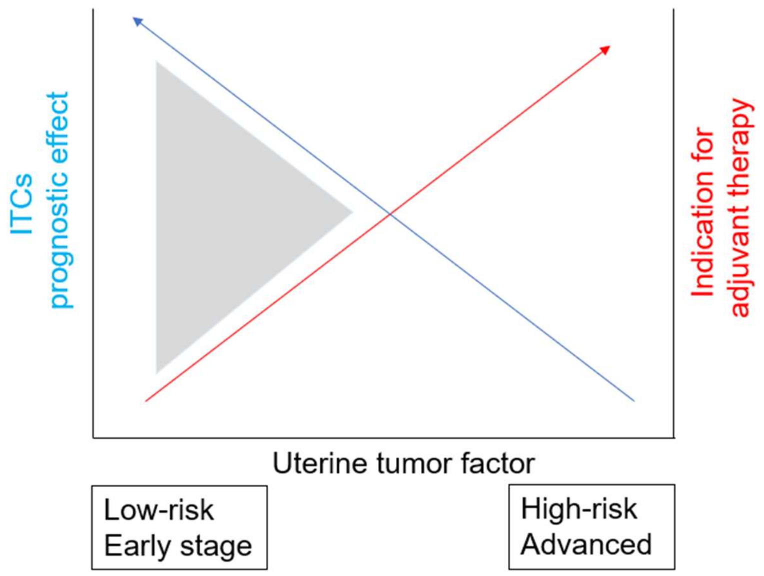

Hypothetical view of prognostic significance of ITCs in endometrial cancer is shown. ITCs in endometrial cancer may be prognostic *(i)* when there is no other uterine factor or *(i)* when uterine factor-tailored adjuvant therapy is not indicated (gray zone). Abbreviation: ITCs, isolated tumor cells.

© 2024 Matsuo K et al. *JAMA Network Open.*
