# Supplementary material for: Lymph Node Isolated Tumor Cells in Patients With Endometrial Cancer
Source: JAMA Netw Open. 2024 Mar 18;7(3):e240988. doi: 10.1001/jamanetworkopen.2024.0988 (PMC10949095; doi:10.1001/jamanetworkopen.2024.0988)
Supplement: Supplement 2. — Data Sharing Statement [file jamanetwopen-e240988-s002.pdf]

Data Sharing Statement

Matsuo. Lymph Node Isolated Tumor Cells in Patients With Endometrial Cancer. *JAMA Netw Open*. Published March 18, 2024. doi:10.1001/jamanetworkopen.2024.0988

Data
Data available: No

Additional Information
Explanation for why data not available: Data sharing statement: The data on which this study is based are American College of Surgeons National Cancer Database (https://www.facs.org/quality-programs/cancer-programs/national-cancer-database/).
